# Respecting the Patient’s Choice: A Case of Possible Drug-Induced Parkinsonism

**DOI:** 10.3390/pharmacy10010010

**Published:** 2022-01-04

**Authors:** Megan R. Undeberg, Kimberly C. McKeirnan, David Easley

**Affiliations:** 1 Pharmacotherapy Department, College of Pharmacy and Pharmaceutical Sciences, Washington State University, Spokane, WA 99202, USA; meganru@wsu.edu; 2 Independent Researcher, Newport, WA 99156, USA; suee@ztc.net

**Keywords:** drug-induced Parkinsonism, rural patient health, integrated medical services, comprehensive medication management

## Abstract

This report describes a case of likely drug-induced Parkinsonism (DIP) identified by the pharmacist. A 54-year-old female patient was referred by a physician to the pharmacist in a rural, integrated care team for a comprehensive medication review (CMR) to address the patient’s concerns of possible Parkinson’s disease (PD). While PD may occur over the progression of age, medications that affect dopamine transport can also cause DIP, a secondary form of Parkinson’s disease. Although PD and DIP may be clinically indistinguishable, differentiation may be possible by reviewing a patient’s medication history for any potential causative drugs correlating to the timeline of the onset of symptoms. In this case, the pharmacist reviewed the medication profile and identified medications that could be responsible for causing DIP, specifically bupropion. The pharmacist suggested discontinuing bupropion and identifying another option for treating depression. The patient appreciated the suggestion and education, but ultimately preferred continuing her bupropion therapy instead of discontinuing therapy or changing to an alternative agent. At a follow-up meeting with the pharmacist, not only was the patient still experiencing tremors despite taking carbidopa/levodopa, but additional medications known to be potential inducers of tremors were added to her regimen. Although the pharmacist repeatedly discussed DIP with the patient and believed stopping bupropion would determine whether her Parkinsonism was PD or DIP, ultimately the patient continued taking bupropion because of concerns related to depression severity and the impact on her well-being. The patient’s wishes were respected.

## 1. Introduction

Parkinson’s disease (PD) is a progressive neurodegenerative disease that presents with tremors, bradykinesia, and rigidity [1]. The patient population generally affected typically increases with age, with the highest incidences between ages of 60 and 80 years due to the natural deterioration of the dopaminergic neurons in the brain over time [2]. While Parkinson’s disease may occur over the progression of age, medications that affect dopamine transport can also induce a secondary, reversible form of Parkinson’s disease called drug-induced Parkinsonism (DIP). Parkinsonism is a general term used for a class of neurological disorders, including Parkinson’s disease, that cause tremors, slow movement, stiffness, and other movement problems [3].

DIP is one of the most common drug-induced movement disorders with an average incidence in the U.S. estimated at 3.3 per 100,000 person-years, making up 11.9% of all cases of parkinsonism [4]. However, DIP is commonly misdiagnosed, making calculation of an exact prevalence rate difficult. DIP can be caused by any medication that interferes with dopamine transmission. DIP symptoms can be misdiagnosed as idiopathic Parkinson’s disease as well as other drug-induced movement disorders such as extrapyramidal symptoms, tardive dyskinesia, and tremors [5]. Although PD and DIP may be clinically indistinguishable, differentiation may be possible by reviewing the patient’s medication history for any potential causative drugs correlating to the timeline of the onset of symptoms [6]. Onset of the symptoms typically occurs within a few weeks to months after initiation of the causative agent. In cases of DIP, if the causative agent is discontinued, the parkinsonism should resolve within six months [6].

PD and DIP disrupt the activities of daily living, can lead to a loss of independence, and can decrease the quality of life for patients. Some problems that may arise throughout the progression of PD and DIP are increasing problems with impaired speech, dysphagia, mobility, and depression. In disease progression, dopamine is depleted from the basal ganglia circuits over time, resulting in disruption in the connection between the thalamus and motor cortex, which leads to the key motor impairment symptoms of PD [7]. Some causative drugs that affect this transport include dopamine D2 receptor-blocking agents such as the antipsychotics, as well as other classes that block D2 receptors such as antiemetics/promotility agents, antidepressants, and calcium channel blockers [8,9]. Such medications are also commonly seen amongst the medication history of older patients for other indications, which puts them at higher risk of possible DIP. Therefore, reviewing the patient’s medication history is important in identifying signs of parkinsonism and differentiating between DIP and PD.

## 2. Materials and Methods

### 2.1. Setting

In a rural, Washington state community, a novel integrated care team including a home health nurse, a hospital pharmacist, and clinic-based physicians developed an interprofessional practice to identify and intervene with high-risk patients cared for in their clinic [10]. Clinic patients who would benefit from an in-depth medication review by the pharmacist were identified by nurses and physicians. Following review and approval by the patient’s primary care provided (PCP), the patients were asked whether they would be interested in meeting with a pharmacist for a comprehensive medication review (CMR), either at a home-based visit or concurrently at an upcoming clinic visit.

Once the patient was enrolled and consented, the pharmacist reviewed the patient’s medication list and electronic health record (EHR) profile to identify possible drug interactions and other medication-related problems and to prepare a list of questions and concerns for a meeting with the patient. 

During the in-person meeting with each patient, the pharmacist completed a CMR session, which included a reconciliation of the actual usage of prescription and non-prescription medications with the patient’s EHR medication list and a discussion of diet, lifestyle, and any concerns the patient wanted to discuss. The pharmacist also obtained a signed waiver allowing him to contact the local community pharmacy for the patient’s prescription refill history to assess medication adherence. 

After the pharmacist met with the patient, he created a summary document with his meeting notes and recommendations. He provided his recommendations and summary to the home health nurse, who entered the information into the EHR for the PCP to review. The PCP would then review the information, address the identified needs, and prioritize next steps including patient education, ordering additional labs or diagnostic tests, and adjusting medications. This project was reviewed and approved by the Washington State University Institutional Review Board. 

### 2.2. Case Presentation

This is the case of a 54-year-old Caucasian woman in a rural Washington State community who lives at home alone. At the time of the visit, she was retired, but she had previously been employed as a nurse and, most recently, she was the primary caregiver for her sister until her passing. She was identified by her primary care provider and home health nurse to be a candidate for pharmacist-provided comprehensive medication review (CMR) services [10]. The patient’s past medical history included diagnoses of angina, asthma, anxiety disorder, chronic neuropathic pain, constipation, depression, diabetes, gastroesophageal reflux disease (GERD), hypertension, insomnia, migraine headaches, muscle spasms, and restless leg syndrome. The patient’s main concern was her development of tremors. From her previous experience as a nurse, the patient reported concerns that she had developed Parkinson’s disease. The primary care provider believed the tremors were due to PD and prescribed a low dose of carbidopa/levodopa. The patient agreed to meet with the pharmacist at one of her upcoming clinic appointments to discuss her medications and possible causes of her tremors.

### 2.3. Pharmacist Intervention

Prior to the face-to-face encounter, the pharmacist reviewed the medication profile of the patient, using the information contained in the patient’s medical record. The pharmacist assessed the 29 current prescription and non-prescription medications that were included in the patient’s EHR profile, displayed in Table 1. He expressed concerns about the complexity of the patient’s medication regimen and reviewed the medications for drug interactions using Drug Facts and Comparisons [11]. Seventeen major drug interactions were identified upon the initial analysis by the pharmacist. These are documented in Table 2. If a medication was noted to have tremors listed as a possible side effect, or drug interactions leading to movement-related symptoms, such as serotonin syndrome, it was documented.

The pharmacist met with the patient in September 2019. She was known to him from previous interactions in his role as a local pharmacist and living in the same small community. During the visit, the pharmacist noted that the patient was not always forthcoming with details and information related to her health, medical conditions, and medications. Due to his frequent previous interactions with the patient during her career as a nurse, the pharmacist expected the patient to communicate in a direct manner with meticulous details. He was surprised when she seemed hesitant to provide insight into her symptoms and avoided conversation related to bupropion.

The pharmacist shared his findings with the patient, including the identification of six possible medications that were known to cause tremors. These included bupropion (28%), albuterol (7–24%), ropinirole (6%), oxycodone (3%), and buspirone (1%) [12]. The pharmacist suggested discontinuing bupropion and identifying other medication options for treating depression. The patient appreciated the suggestions and education about her medications, but ultimately preferred continuing her bupropion therapy instead of discontinuing therapy or changing to an alternative agent. She reported having difficulty finding a medication that controlled her severe depression and did not want to switch now that it was more controlled. She reported that her depression had previously impacted her quality of life significantly and shared with the pharmacist that other medications for depression she had tried did not markedly improve her symptoms. Additional details regarding which medications she had previously taken and reasons for discontinuation were not disclosed by the patient. 

After meeting with the patient, the pharmacist provided a summary of his visit and his recommendations as written chart notes and verbally via direct provider-to-provider conversation with the patient’s physician. The pharmacist reported his concerns that bupropion may be causing DIP but that the patient was not interested in discontinuing the medication. The family physician confirmed he was aware of the risk of possible DIP and agreed to meet with the patient again to discuss this with her and conduct another assessment for PD. 

The physician later met with the patient for a follow-up visit. He reported confirming the diagnosis of PD and choosing to respect the patient’s wishes to continue taking bupropion despite the known possibility it could be causing or worsening her tremors. To better manage the tremors, he also increased the carbidopa/levodopa to 25 mg/100 mg, two tablets by mouth four times daily. 

## 3. Results

Between September 2019 and March 2020, the patient continued to see her physician. During this time, the patient’s medication profile expanded further to incorporate additional therapies prescribed to better manage respiratory conditions (COPD and allergic rhinitis), GERD (addition of promotility agent of domperidone, imported via mail order from Canada), and rapid upward titration of carbidopa and levodopa for management of the tremor. The tremor had not abated, even with the addition of the extended-release formulation of carbidopa/levodopa as well as increased doses of the immediate-release carbidopa/levodopa. Medications that were added to the patient’s profile between September 2019 and March 2020 are displayed in Table 3.

At the request of the physician, the pharmacist engaged the patient in a clinic-based follow-up appointment in March 2020. She was counseled again regarding the possibility of having DIP caused by her bupropion. She again declined any additional interventions to discontinue or alter her bupropion use. The pharmacist determined that three of the new medications added to her profile were also known to cause tremors. The new medications along with the incidence that each causes tremor is displayed in Table 4.

## 4. Discussion

Parkinsonism movement symptoms present in a variety of ways, including slowness, stiffness, resting tremor, and changes in gait and balance. When asked to perform an action, the tremor will subside, only to be noted again when the patient’s limb is at rest. DIP is typically caused by the blockade on the dopamine receptors, which mimics what occurs in Parkinson’s disease when a patient experiences loss of dopaminergic neurons in the brain [13]. A summary of medications known to cause drug-induced Parkinsonism, including atypical and typical antipsychotics, many anti-depressants, and anti-nausea medications, is shown in Table 5 [14,15,16,17,18,19,20,21,22,23]. The risk is often dose dependent, and typically resolves within six months when the offending agent is identified and discontinued [24].

Tremor, whether caused by a disease or as a side effect of a medication, can be distressing to a patient. In this clinical case presentation, the patient’s tremulous symptoms seemed to be related to the introduction of bupropion to her medication regimen. Bupropion has a risk of inducing tremor (28%) [12]. Despite identification of the possible DIP by the pharmacist and repeated suggestions to discontinue the medication with the greatest incidence of tremors, the patient opted to continue her current medication regimen, including bupropion. At the follow-up meeting with the pharmacist, not only was the patient still experiencing tremors despite the upward titration to a very high dose of carbidopa/levodopa, but she had also started taking other potential inducers of tremors. Although the pharmacist repeatedly discussed DIP with the patient, ultimately it was her decision. 

Patient engagement is essential for shared decision making among the patient, pharmacist, and physician. Shared decision making is a model involving the patient and providers working together and has been previously utilized for treatment of depression [25,26,27]. According to the Agency for Healthcare Research and Quality (AHRQ), there are five principles of shared decision making: seeking the patient’s participation; helping the patient explore options; assessing the patient’s values and preferences; reaching a decision with the patient; and evaluating the patient’s decision [25]. All five elements of the AHRQ model were incorporated into this case and the patient was engaged and well informed about her options. Although interprofessional shared decision making involves providers supporting one another’s expertise, in this case the patient’s preferences regarding bupropion were ultimately the deciding factor in continuation of the therapy.

The advantage of an integrated care team is to unite and coordinate care for at-risk patients and lay the groundwork for shared decision making. The role of the pharmacist is to advance knowledge, identify potential risks, and work with the patient and care team to meet goals. Here, the pharmacist was able to identify multiple viable sources of drug-induced tremor in the patient; the challenge was when the patient opted to not pursue any changes. Follow-up ensued, at which time the pharmacist was able to note continued worsening of the patient as well as an increase in targeted medication use. These brief, clinic-based interactions encourage continued integrated care, shared decision making and patient-centered outcomes.

## 5. Conclusions

DIP and PD are virtually indistinguishable unless the causative agent is removed to determine whether symptoms improve. DIP is reversible but only when the patient is willing to discontinue the medication that is thought to be causing DIP. In this case, shared decision making prioritized the patient’s preference for treating her depression over addressing the possible DIP. Her health care providers respected her wishes. 

## Figures and Tables

**Table 1:** The patient’s initial medication list organized by indication.

Medical Indication	Medication and Regimen
Angina	Nitroglycerin SL 0.4 mg sublingually as needed for angina
Anxiety disorder	Buspirone 30 mg by mouth two times daily Clonazepam 0.5 mg by mouth two times daily as needed for anxiety
Asthma	Albuterol 0.5% nebulizer solution OR albuterol MDI 90 mcg actuation (2 puffs); inhalation of either every 6 h as needed for shortness of breath Montelukast 10 mg by mouth once daily Pulmicort 180 mcg/actuation inhaled two times daily
Depression	Bupropion ER 200 mg by mouth two times daily
Diabetes	Lantus Solostar 100 units/mL 30 units injected subcutaneously two times daily (in the morning and evening) Metformin 1000 mg by mouth two times daily Novolog Flexpen 100 units/mL 2–10 units injected subcutaneously by sliding scale before meals and at bedtime if having bedtime snack
GERD	Omeprazole 20 mg by mouth two times daily Ranitidine 150 mg by mouth two times daily
Hyperlipidemia	Simvastatin 40 mg by mouth every night at bedtime
Hypertension	Hydrochlorothiazide 25 mg by mouth daily Lisinopril 2.5 mg by mouth daily
Insomnia	Suvorexant 20 mg by mouth every night at bedtime
Migraine headaches	Eletriptan 40 mg tab by mouth as needed for headache
Nausea, vomiting	Ondansetron 8 mg by mouth three times daily as needed for nausea
Pain	Gabapentin 800 mg by mouth three times daily Morphine sulfate ER 15 mg by mouth two times daily Oxycodone/APAP 10/325 mg by mouth four times daily
Muscle spasm	Tizanidine 2 mg by mouth every 8 h
Parkinson’s disease	Carbidopa/Levodopa 25/100 mg by mouth three times daily
RLS	Ropinirole 0.25 mg by mouth two times daily Magnesium oxide 400 mg by mouth daily
Supplementation	Multivitamin by mouth daily Vitamin D3 1000 international units by mouth daily

GERD: gastroesophageal reflux disease; MDI: metered dose inhaler; RLS: restless leg syndrome; ER: extended release; SL: sublingual.

**Table 2:** Drug interactions identified for this patient [12].

Interacting Medications	Resulting Effect
oxycodone/APAP and ranitidine	Increased oxycodone level
oxycodone/APAP and tizanidine	Increased risk of paralytic ileus Increased risk of respiratory and central nervous system depression
budesonide and bupropion ER	Risk of lowering the seizure threshold
bupropion ER and ranitidine	Reduced renal clearance of ranitidine; increased risk of ranitidine adverse drug events
**bupropion ER and carbidopa/levodopa**	**Concurrent use of bupropion and levodopa may result in CNS toxicity, including tremor**
buspirone and gabapentin	Increased risk of respiratory depression
**buspirone and morphine**	Increased risk of respiratory and CNS depression **Increased risk of serotonin syndrome**
**buspirone and ondansetron**	**Increased risk of serotonin syndrome**
**buspirone and hydrocodone/APAP**	Increased risk of respiratory and CNS depression **Increased risk of serotonin syndrome**
clonazepam and gabapentin	Increased risk of respiratory depression
clonazepam and oxycodone/APAP	Increased risk of respiratory and central nervous system depression
clonazepam and morphine	Increased risk of respiratory and central nervous system depression
**eletriptan and morphine**	**Increased risk of serotonin syndrome**
**eletriptan and ondansetron**	**Increased risk of serotonin syndrome**
**eletriptan and oxycodone/APAP**	**Increased risk of serotonin syndrome**
gabapentin and oxycodone/APAP	Increased risk of respiratory depression
gabapentin and tizanidine	Increased risk of respiratory depression
**morphine and ondansetron**	**Increased risk of serotonin syndrome**
morphine and suvorexant	Increased risk of respiratory and central nervous system depression
morphine and oxycodone/APAP	Increased risk of respiratory and central nervous system depression
morphine and tizanidine	Increased risk of paralytic ileus Increased risk of respiratory and central nervous system depression
ondansetron and bupropion	Increased level of bupropion; may need dose adjustment
**ondansetron and oxycodone/APAP**	**Increased risk of serotonin syndrome**
**suvorexant and oxycodone/APAP**	**Increased risk of serotonin syndrome**

Bold indicates drug interactions leading to movement-related symptoms, including tremor. CNS: central nervous system; APAP: acetaminophen.

**Table 3:** Medication changes initiated between September 2019 and March 2020.

Indication	Medication Added, Frequency
Allergic rhinitis	Fluticasone propionate 50 mcg/spray, 2 sprays in each nostril daily as needed
Asthma/COPD	Ipratropium/albuterol 0.5 mg/ 3 mg nebulizer solution, 1 vial in nebulizer every six hours
Pain, anti-inflammatory	Naproxen 500 mg by mouth two times daily
Parkinson’s disease	Carbidopa/Levodopa 25/100 mg, 2 by mouth 4 times daily
Parkinson’s disease	Carbidopa/Levodopa ER 50 mg/ 200 mg, 2 at 7am, 1 at 11 am, 1 at 3 pm, and 2 at 7 pm
GERD	Domperidone 10 mg by mouth three or four times daily before meals (imported from Canada)
Neuropathic pain	Pregabalin 150 mg by mouth two times daily
**Indication**	**Medications Discontinued, Frequency**
Anxiety disorder	Clonazepam 0.5 mg by mouth two times daily as needed for anxiety
Neuropathic pain	Gabapentin 800 mg by mouth three times daily

**Table 4:** Medications added to the patient’s medication regimen that were associated with tremor, as noted with follow-up visit by pharmacist [12].

Medication	Possible Tremor-Related Effect
Domperidone	Risk of tremor and EPS symptoms, percent unspecified
Pregabalin	Tremor: 1% to 11.2%
Albuterol	Tremor: 5% to 7% or more

**Table 5:** Drug classes known to cause drug-induced parkinsonism with risk level [14,15,16,17,18,19,20,21,22,23]. Adapted from [14].

Drug Class	Example Medications	Level of Risk
Antipsychotics	chlorpromazine, thioridazine, perphenazine, fluphenazine, thiothixene, pimozide, loxapine, amoxapine, haloperidol	High
Atypical Antipsychotics	olanzapine, risperidone, aripiprazole	High
	quetiapine, clozapine	Low
Neuroleptics	prochlorperazine, promethazine, hydroxyzine, metoclopramide, cisapride	Intermediate to High
Tricyclic Antidepressants	amitriptyline, imipramine, clomipramine	Intermediate
Selective Serotonin Reuptake Inhibitors	fluoxetine, fluvoxamine, sertraline, mirtazapine, paroxetine, citalopram, escitalopram	Intermediate
Selective Serotonin Reuptake Inhibitors	fluoxetine, fluvoxamine, sertraline, mirtazapine, paroxetine, citalopram, escitalopram	Intermediate
Mood stabilizers	lithium	Intermediate
Anticonvulsants	phenytoin, valproic acid	Intermediate
Prokinetic Agents	domperidone	Low
Calcium Channel Blockers	diltiazem, verapamil	Low
Monoamine Oxidase Inhibitors	phenelzine	Low
Antiarrhythmics	amiodarone	Low
Immunosuppressants	cyclosporine	Low
Antivirals	acyclovir, vidarabine, antiretrovirals	Low
Antifungals	amphotericin B	Low
Chemotherapy	thalidomide	Low
Endocrine Hormones	levothyroxine	Low
